# Midterm Outcomes of Graft Insertion Technique for Redo Aortic Root Surgery

**DOI:** 10.5761/atcs.oa.25-00047

**Published:** 2025-07-10

**Authors:** Takuya Narita, Ai Ishizawa, Nobuyuki Inoue, Tetsuro Uchida, Yoshitsugu Nakamura

**Affiliations:** 1 Cardiovascular Surgery, Chiba-Nishi General Hospital, Matsudo, Chiba, Japan; 2 Cardiovascular Surgery, Yokohama Rosai Hospital, Yokohama, Kanagawa, Japan; 3 The Second Department of Surgery, Yamagata University Hospital, Yamagata, Yamagata, Japan; 4 Cardiovascular Surgery, National Center for Global Health and Medicine, Tokyo, Japan

**Keywords:** graft insertion technique, aortic root abscess, endocarditis, inversion technique

## Abstract

**Purpose:** This study evaluated the midterm outcomes, including adverse aortic events (AAEs), of the “graft insertion technique” (GIT) for left ventricular outflow tract (LVOT) and aortic root reconstruction.

**Methods:** From August 2014 to March 2024, 14 consecutive patients underwent GIT for LVOT and aortic root reconstruction. The indications for surgery were prosthetic valve endocarditis in 9 cases and noninfectious pseudoaneurysm in 5 cases. Among these patients, seven (50.0%) underwent aortic root surgery, while the other seven (50.0%) underwent aortic valve replacement alone or in combination with other procedures without aortic root surgery. Their mean EuroSCORE II was 28.8 ± 17.6.

**Results:** The mean total operation time was 504 ± 87 min. The mean cardiopulmonary bypass and aortic cross-clamp times were 311 ± 41 and 240 ± 45 min, respectively. Operative mortality occurred in one case (7.1%), and five patients (35.7%) died during the first year of follow-up. No surviving patients experienced recurrent endocarditis. No patients died from cardiovascular events or infections after the second year of follow-up. Furthermore, no AAEs were observed on computed tomography during the follow-up period after hospital discharge.

**Conclusion:** GIT is a feasible alternative for high-risk cases of redo aortic root surgery.

## Introduction

Reoperations for aortic valve and aortic root replacements due to infective endocarditis (IE) are challenging.^1)^ The annual incidence of native valve endocarditis (NVE) is estimated at 5.0–7.9 episodes per 100000 patient-years, while prosthetic valve endocarditis (PVE) occurs at a rate of 0.2–1.4 episodes per 100 patient-years.^2)^ A recent report from the Society of Thoracic Surgeons database indicated an increase in the incidence of PVE from 20.9% in 2011 to 25.9% in 2019.^3)^ This report also noted that PVE is associated with higher operative mortality and morbidity compared to NVE. Additionally, aortic root abscesses are more common in PVE (50.1%) than in NVE (25.2%), and such abscesses are considered a significant risk factor for mortality and morbidity.^4–6)^

The primary goal of surgery for PVE is to control the infection by removing foreign materials and debriding infected tissues. Subsequent procedures include conventional aortic valve replacement (AVR), patch repair with AVR, or aortic root surgery.^7,8)^ In cases with significant damage or defects in the area between the left ventricular outflow tract (LVOT) and aortic root, aortic root surgery becomes necessary. However, when aortic root replacement is required due to IE, the surgical outcomes are often poor. Heubner et al. reported that nearly half of the 130 patients undergoing aortic root reoperation had IE, with the 2nd operations performed by their team including stentless bioprosthesis implantation, as well as the Bentall (mechanical valve), Ross, and valve-sparing procedures. Notably, outcomes for IE cases were significantly worse than those for non-IE cases.^9)^ Another concern is the formation of pseudoaneurysms after reoperative aortic root surgery, which is associated with vulnerable tissue. However, there is limited focus on adverse aortic events (AAEs) in relation to aortic root surgery.^10)^

Nakamura et al.^11)^ proposed the graft insertion technique (GIT) using an artificial tube graft as a salvage method for reconstructing damaged LVOT and aortic root. In this study, we summarized the midterm outcomes of GIT and the occurrence of AAEs on computed tomography (CT).

## Materials and Methods

### Study population

From August 2014 to March 2024, 14 consecutive patients underwent redo LVOT reconstruction and aortic root surgery using GIT at Chiba-Nishi General Hospital, Chiba, Japan, and Yamagata University Hospital, Yamagata, Japan. Data were retrospectively collected from the databases of both institutions.

**Table 1** summarizes the patients’ demographic and clinical characteristics. Eleven patients were enrolled from Chiba-Nishi General Hospital, and 3 patients were from Yamagata University Hospital. The cohort consisted of 13 men (92.9%) and one woman (7.1%), with a mean age of 64.6 ± 13.4 years. The average New York Heart Association classification score and EuroSCORE II were 2.0 ± 0.9 and 28.8 ± 17.6, respectively. For the primary surgery, 7 patients (50.0%) underwent aortic root surgery, including the Bentall and David procedures, while the remaining 7 patients (50.0%) underwent AVR alone or in combination with other procedures without aortic root surgery. The indication for redo surgery was infection in 9 patients (64.3%) and noninfectious pseudoaneurysm in 5 cases (35.7%).

**Table 1:** Preoperative patient characteristics

Characteristics	Overall (N = 14)
Age (years)	64.6 ± 13.4
Sex, male (no. [%])	13 (92.9)
BSA (m^2^)	1.6 ± 0.2
NYHA classification	2.0 ± 0.9
EuroSCORE II (%)	28.8 ± 17.6
CHF after primary surgery (no. [%])	0 (0)
SSS or complete AV block (no. [%])	5 (35.7)
Hypertension (no. [%])	10 (71.4)
Dyslipidemia (no. [%])	6 (42.9)
Diabetes (no. [%])	3 (21.4)
Stroke (no. [%])	6 (42.9)
Coronary artery disease (no. [%])	3 (21.4)
PAD (no. [%])	1 (7.1)
COPD (no. [%])	2 (14.3)
ESRF on dialysis (no. [%])	0 (0)
Cirrhosis (no. [%])	1 (7.1)
Steroid user (no. [%])	0 (0)
Emergency (no. [%])	5 (35.7)
Shock (no. [%])	0 (0)
Primary surgery	
Bentall (no. [%])	3 (21.4)
Bentall + CABG (no. [%])	1 (7.1)
Bentall + TAR (no. [%])	2 (14.3)
David + TAR (no. [%])	1 (7.1)
AVR (no. [%])	4 (28.6)
AVR + AAR (no. [%])	1 (7.1)
AVR + MV repair + maze procedure + LAAC (no. [%])	1 (7.1)
AVR + TV annuloplasty + VSD closure (no. [%])	1 (7.1)
Indication for secondary cardiac surgery	
Infection (no. [%])	9 (64.3)
Native tissue (no. [%])	0 (0)
Prosthetic valve/graft (no. [%])	9 (64.3)
Noninfectious pseudoaneurysm (no. [%])	5 (35.7)

AAR: ascending aorta replacement; AV: atrioventricular; AVR: aortic valve replacement; BSA: body surface area; CABG: coronary artery bypass grafting; CHF: congestive heart failure; COPD: chronic obstructive pulmonary disease; ESRF: end-stage renal failure; LAAC: left atrial appendage closure; MV: mitral valve; NYHA: New York Heart Association; PAD: peripheral arterial disease; SSS: sick sinus syndrome; TAR: total arch replacement; TV: tricuspid valve; VSD: ventricular septal defect

### Surgical technique

**Table 2** presents the descriptions of each surgical finding leading to GIT.

**Table 2:** Operative findings in 14 patients

Cases	Pathogens	Operative findings	Comments
1	CNS	1) PVE	1) Debridement extended to MV
		2) Abscess cavity at the membranous portion	→ MVR
			2) Debridement created cavity communication from LVOT to RA and RV
			→ Direct suture closure
2	Staphylococcus aureus	1) PVE	
		2) Peri-artificial graft abscess	
3	MRSA	1) PVE	Debridement created communication from LVOT to RA
		2) Aortic annular abscess at RCC and NCC	
		3) Detachment of the previous AV	→ Patch closure
4	Streptococcus species	1) PVE	
		2) Circumferential annular abscess	
5	CNS	1) PVE	
		2) Peri-artificial graft abscess	
		3) Pseudoaneurysm at previous proximal anastomosis	
		4) Detachment of the previous composite graft	
6	N/A	1) Pseudoaneurysm at previous proximal anastomosis	
		2) Detachment of the previous composite graft	
7	N/A	1) Pseudoaneurysm at LCC-RCC commissure of the previous anastomosis	
		2) Detachment of the previous AV	
8	Streptococcus species	1) PVE	
		2) Aortic annular abscess at the RCC	
9	N/A	1) Aortic root dilation	Aortic annulus was impossible to stitch.
		2) Severely calcified aortic annulus	
10	Enterococcus faecalis	1) PVE	
		2) Pseudoaneursym at the LCC	
		3) Circumferential annular abscess	
11	MRCNS	1) PVE	
		2) Peri-artificial graft abscess	
		3) Pseudoaneurysm at previous proximal anastomosis	
		4) Detachment of the previous composite graft	
12	N/A	1) Pseudoaneurysm	
		2) Detachment of the previous freestyle conduit at the LCC and NCC	
13	Enterococcus faecalis	1) Pseudoaneurysm	The perforation was sewn together with the new inverted graft.
		2) Extracardiac perforation at subaortic curtain	
		3) Post-omental transfer for mediastinitis	
14	N/A	1) Pseudoaneurysm	
		2) Detachment of the previous artificial graft for the David procedure at the RCC and NCC	

AV: aortic valve; CNS: coagulase-negative staphylococci; LCC: left coronary cusp; LVOT: left ventricular outflow tract; MRCNS: methicillin-resistant coagulase-negative staphylococci; MRSA: methicillin-resistant *Staphylococcus aureus*; MV: mitral valve; MVR: mitral valve replacement; N/A: not available; NCC: noncoronary cusp; PVE: prosthetic valve endocarditis; RA: right atrium; RCC: right coronary cusp; RV: right ventricle

**Fig. 1:**
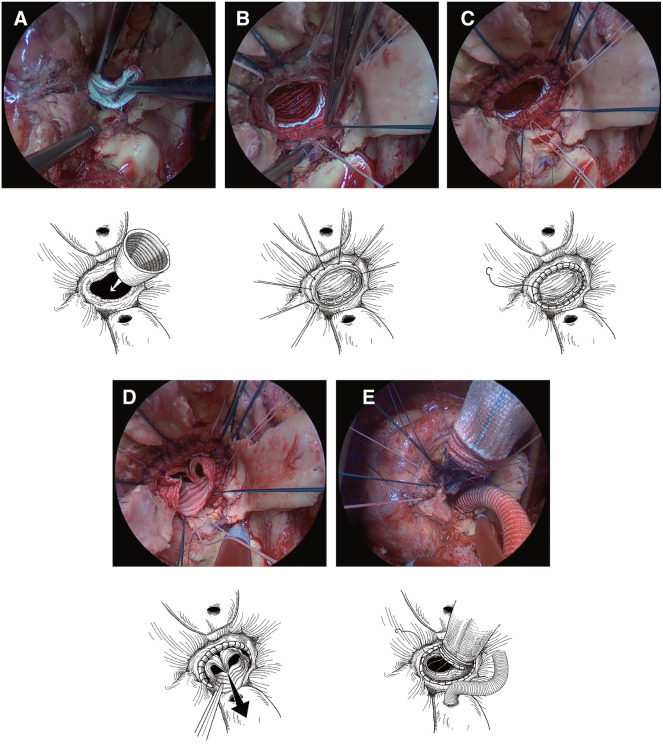
Comparative analysis between operative images and schemas of the graft insertion technique. (**A)** A 3-cm inverted straight graft, matching the diameter of the measured LVOT, was inserted into the LVOT. (**B)** Nine to 12 horizontal mattress sutures were placed from inside the graft to the epicardial side through Teflon felt using 2-0 synthetic braided and pledgeted sutures. (**C)** The seam was reinforced with 4-0 polypropylene running over-and-over sutures at the edges of the LVOT and graft. (**D)** The inserted tube graft was pulled out of the LVOT. (**E)** The new LVOT was trimmed short, and the composite graft for the Bentall procedure was anastomosed to the new LVOT with a running suture. Coronary buttons were reconstructed using the Carrel patch technique and/or Piehler’s technique. LVOT: left ventricular outflow tract

GIT was performed according to the procedures described previously (**Fig. 1** and **Supplementary Movie**).^11)^ Briefly, after performing a secondary median sternotomy, the aortic root was dissected and exposed. Subsequently, cardiopulmonary bypass was initiated in the standard manner. Cardiac arrest was achieved by clamping the ascending aorta. The prosthesis was removed from the aortic root, and the affected tissue was extensively debrided. Notably, depending on the extent of debridement associated with suturing during LVOT and aortic root reconstruction described below, avoiding atrioventricular block may not be possible. Next, the coronary buttons were prepared. A 3-cm inverted straight graft with a diameter matching that of the measured LVOT was inserted into the LVOT (**Fig. 1A**). From inside the inserted graft to the epicardial side through Teflon felt, 9–12 horizontal mattress sutures were placed in as much healthy tissue as possible at the LVOT using 2-0 synthetic braided and pledgeted sutures. It is important to note the length and thickness of the felt. Otherwise, this may cause narrowing of the LVOT (**Fig. 1B**). The seam was reinforced with 4-0 polypropylene running over-and-over sutures along the edges of the LVOT and graft (**Fig. 1C**). The straight graft was then pulled out (**Fig. 1D**), exposing its right side. The new LVOT was trimmed to the desired length, and the composite graft for the Bentall-type operation was anastomosed to the new LVOT with a running suture. Coronary buttons were reconstructed using the Carrel patch technique and/or Piehler’s technique (**Fig. 1E**).

### Data collection and statistical analysis

Perioperative data, along with follow-up information after hospital discharge, were collected from the computerized databases of our hospital, family physicians, and patients or their close family members (via telephone). Follow-up was completed for all patients; however, some data were missing, as detailed in the Results section. Categorical variables are presented as counts and percentages, whereas continuous variables are expressed as means ± standard deviations. All statistical analyses were performed using EZR (Saitama Medical Center, Jichi Medical University, Saitama, Japan), which is a graphical user interface for R (The R Foundation for Statistical Computing, Vienna, Austria). More precisely, it is a modified version of R Commander designed to add statistical functions frequently used in biostatistics.

## Results

**Table 3** summarizes the operative data. All operations were redo cases involving GIT for LVOT reinforcement and the Bentall procedure. The mean cardiopulmonary bypass and aortic cross-clamp times were 311 ± 41 and 240 ± 45 min, respectively. The mean total operation time was 504 ± 87 min. Circulatory arrest was required in 2 cases (mean duration 39 ± 13 min), with antegrade selective cerebral perfusion performed for brain protection (mean duration 40 ± 13 min). The mean sizes of the graft and valve used in GIT were 25 ± 1 and 21 ± 1 mm, respectively. A tissue valve was used in 11 cases (78.6%). The additional procedures included coronary artery bypass grafting and pulmonary artery repair for a pulmonary fistula in one case each, and mitral and tricuspid valve surgery in another case. The mean total blood loss was 3633 ± 3272 mL.

**Table 3:** Procedural data for operation using the graft insertion technique

Variable	Overall (N = 14)
Total operation time (min)	504 ± 87
Cardiopulmonary bypass time (min)	311 ± 41
Cross-clamp time (min)	240 ± 45
Circulatory arrest time (min)^a)^	39 ± 13
Antegrade selective cerebral perfusion time (min)^a)^	40 ± 13
Graft size (mm)	25 ± 1
Valve size (mm)	21 ± 1
Tissue valve (no. [%])	11 (78.6)
Concomitant procedures (no. [%])	3 (21.4)
CABG (no. [%])	1 (7.1)
PA repair (no. [%])	1 (7.1)
MVR + TV repair (no. [%])	1 (7.1)
Total blood loss (mL)	3633 ± 3272
Mechanical circulatory support (no. [%])	0 (0)

^a)^N = 2.

CABG: coronary artery bypass grafting; MVR: mitral valve replacement; PA: pulmonary artery; TV: tricuspid valve

**Fig. 2:**
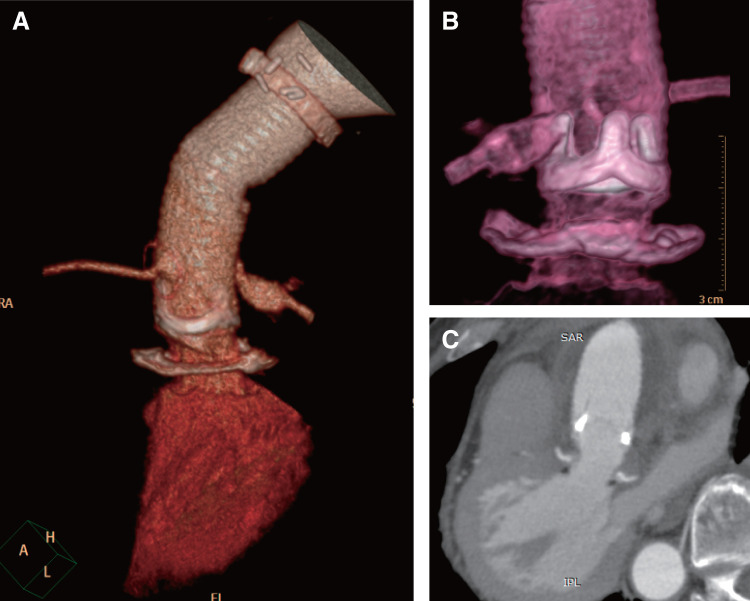
Computed tomography of the LVOT and aortic root post-graft insertion technique. (**A)** 3D reconstruction of the LVOT and aortic root. (**B)** Transparent composite graft. (**C**) No stenosis was observed from the LVOT to the aortic root. LVOT: left ventricular outflow tract

**Figure 2A** shows the general visual appearance of the surgical site, whereas **Fig. 2B** shows the aortic valve through the composite graft via CT. The pressure gradient at the LVOT was not measured by echocardiography; however, postoperative imaging confirmed no LVOT stenosis (**Fig. 2C**).

**Table 4** presents the clinical outcomes. Operative mortality occurred in 1 case (7.1%) due to bleeding on postoperative day 5. Another patient required re-exploration for bleeding on postoperative day 1. No neurological events were observed during the postoperative hospital stay. In addition, no case required mechanical circulatory support postoperatively. However, 3 patients (21.4%) developed new-onset complete atrioventricular block and required permanent pacemaker implantation. The mean duration of mechanical ventilation was 4 ± 5 days, and no tracheostomy was required. Four patients (28.6%) developed acute kidney injury, with 1 patient (7.1%) requiring temporary dialysis. No gastrointestinal bleeding was observed. The mean hospital stay was 53 ± 26 days, and the mean intensive care unit stay was 10 ± 6 days. Postoperative echocardiography during the hospital stay revealed a mean ejection fraction of 55.9% ± 9.2%, a mean pressure gradient of 6.4 ± 3.9 mmHg, and a mean peak aortic velocity of 1.6 ± 0.5 m/s, with no cases of more than moderate aortic insufficiency (**Table 5**). Additionally, no accelerated blood flow was detected at the LVOT below the aortic valve.

**Table 4:** Clinical outcomes

Outcome	Overall (N = 14)
Early^a)^	
Operative mortality	1 (7.1)
Bleeding (no. [%])	1 (7.1)
Re-exploration for bleeding (no. [%])	1 (7.1)
Neurological events (no. [%])	0 (0)
Mechanical circulatory support (IABP, ECMO) (no. [%])	0 (0)
New-onset complete AV block (no. [%])	3 (21.4)
Mechanical ventilation support (days) ^b)^	4 ± 5
Tracheotomy (no. [%])	0 (0)
New-onset acute kidney injury (no. [%])	4 (28.6)
Dialysis (no. [%])	1 (7.1)
GI bleeding (no. [%])	0 (0)
Hospital stay (days)^b)^	53 ± 26
ICU stay (days)^b)^	10 ± 6
Late^c)^	
Death (no. [%])	6 (42.9)
1st Postoperative year	
Acute subdural hematoma (no. [%])	1 (7.1)
Pyogenic spondylitis (no. [%])	1 (7.1)
Heart failure (no. [%])	1 (7.1)
Stroke (no. [%])	1 (7.1)
Unknown (no. [%])	1 (7.1)
2nd Postoperative year onward	
Acute myeloid leukemia (no. [%])	1 (7.1)
PVE (no. [%])	0 (0)
SVD (no. [%])	0 (0)
Development of complete AV block (no. [%])	0 (0)
Adverse aortic events (no. [%])	0 (0)

^a)^During hospitalization after the operation.

^b)^N = 13. One patient died on postoperative day 5.

^c)^After hospital discharge.

AV: atrioventricular; ECMO: extracorporeal membrane oxygenation; GI: gastrointestinal; IABP: intra-aortic balloon pump; ICU: intensive care unit; PVE: prosthetic valve endocarditis; SVD: structural valve deterioration

**Table 5:** Postoperative echocardiographic data before hospital discharge

Parameter	Overall (N = 13)^a)^
Ejection fraction (%)	55.9 ± 9.2
Mean pressure gradient (mmHg)	6.4 ± 3.9
Peak aortic valve velocity (m/s)	1.6 ± 0.5
Moderate or severe aortic regurgitation (no. [%])	0 (0)

^a)^One patient died on postoperative day 5 before echocardiography could be performed.

**Fig. 3:**
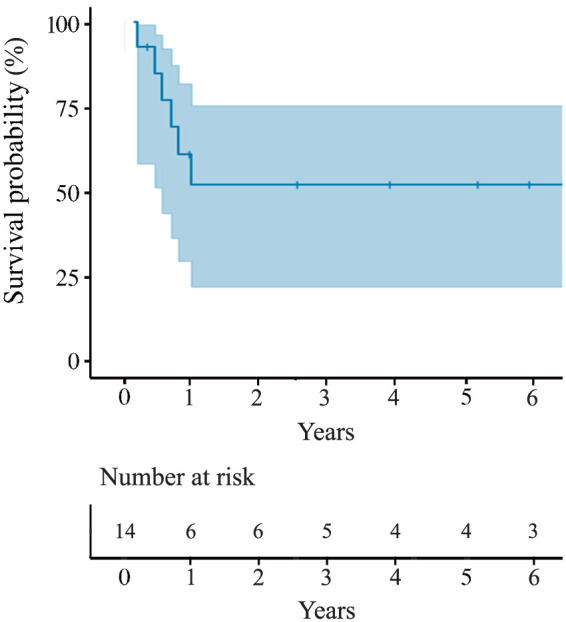
Kaplan–Meier actuarial survival curve for patients who underwent the graft insertion technique for left ventricular outflow tract and aortic root reconstruction.

During the follow-up period, 6 patients (42.9%) died: one from acute subdural hematoma (postoperative day 112), one from sepsis due to pyogenic spondylitis (postoperative day 156), one from heart failure (postoperative day 209), one from cerebral infarction (postoperative day 244), one from unknown causes (postoperative day 320), and one from acute myeloid leukemia (6th postoperative year). No cases of recurrent PVE, prosthetic valve failure (PVF), or new-onset complete atrioventricular block were observed after hospital discharge (**Table 4**). Kaplan–Meier analysis revealed a 53.1% chance of survival at one year, which remained constant at 3 and 5 years (**Fig. 3**). It is notable that no evidence of AAEs, including those among deceased cases, was detected on CT during the observation period (**Table 4**).

## Discussion

This study evaluated the effectiveness and feasibility of the GIT for redo surgery in high-risk patients with extensive defects in the LVOT and aortic root. Generally, operative mortality in reoperations can be influenced by factors such as the primary surgery, preoperative patient condition, and the complexity of the redo procedure.^12–14)^ Infection poses one of the most significant challenges, as infected tissue requires thorough debridement to eliminate bacteria. Moreover, the remaining tissue may be damaged and prone to tearing, and the annular tissue often proves insufficient for anastomosis. Therefore, effective LVOT and aortic root reconstruction are crucial.

Regarding aortic root reconstruction, several studies have explored the use of freehand implantation techniques with aortic root homograft.^15,16)^ Sabik et al.^17)^ reported that homograft offers flexibility, allowing it to be tailored to conform to the LVOT distorted by infection and redo procedures. Additionally, homograft has a relatively high biological resistance to infection. Notably, the remaining anterior mitral leaflet can be used to repair damaged tissues such as fistulas, ventricular septal defects, and abscesses. Despite the promising outcomes reported for freehand aortic homograft implantation, there is a lack of studies specially addressing AAEs in the context of damaged LVOT and aortic root.^18–20)^

The use of homograft has several drawbacks, including tissue damage from manipulation for inversion, postoperative aortic insufficiency, calcification over time, and limited availability.^21)^ To address the issue of homograft scarcity, stentless aortic bioprostheses can be considered; however, reports on their use for aortic valve endocarditis have shown variable follow-up results.^22,23)^ Schneider et al.^22)^ used stentless bioprostheses for aortic valve endocarditis, noting that 54% of their cases had undergone previous AVR. They reported early and late mortality rates of 11% and 14%, respectively. Schaefer et al.^23)^ compared outcomes of stentless bioprostheses for NVE with stented bioprostheses, indicating that valve explantation due to structural degeneration or PVE was more frequent in the stentless bioprostheses group. Subgroup analysis revealed impaired survival outcomes for the stentless prosthetic valve group compared to the stented valve group. Moreover, Hiremath et al.^24)^ applied the inversion technique to stentless bioprostheses; however, follow-up results, including AAEs in the aortic root, have yet to be reported.

While the aforementioned techniques offer excellent surgical visualization and ample working space, GIT presents several additional benefits. First, unlike homografts and stentless bioprostheses, the polyester conduit used in GIT is less prone to damage. Second, prosthesis–patient mismatch can be avoided because the prosthesis size is defined by the composite graft rather than the graft insertion. Third, GIT is technically simpler to perform. Fourth, graft availability is not an issue, allowing surgeons to select appropriately sized grafts.

Masetti et al.^25)^ and Bakhtiary et al.^26)^ reported techniques similar to ours for LVOT and aortic root reconstruction. Their approaches differ from ours in that they use an inverted polyester graft anastomosed to the LVOT with a continuous suture, similar to stentless bioprosthesis inversion techniques. This method may have several concerns. First, a simple linear suture line may be prone to bleeding and pseudoaneurysm formation due to suture dislodgement in fragile tissue. In contrast, our technique uses mattress sutures combined with a continuous suture, creating a more robust and hemostatic plane. In addition, the use of horizontal mattress sutures in our technique allows for more even and precise placement of sutures, accommodating irregularities in the tissue.

Kouchoukos et al.^27)^ followed Masetti’s technique and reported actuarial survival rates at 1, 5, and 10 years of 86.7%, 82.2%, and 62.6%, respectively. However, one patient required reoperation due to pseudoaneurysm formation. Additionally, their study did not include preoperative risk assessment, despite focusing solely on redo cases. El-Sayed Ahmad et al.^28)^ reported on Bakhtiary’s follow-up data, noting 1- and 4-year mortality rates were 34.1% and 39.0%, respectively. The Kaplan–Meier survival estimate was 60.3% at 3 years. However, they did not specify the frequency of AAEs, including pseudoaneurysm formation, within their cohort. Notably, their study included a mixture of redo and primary cases, with redo cases comprising 70.7% of the total.

In contrast, our cohort included redo cases only, and the mean EuroSCORE II was 28.8 ± 17.6, with an operative mortality rate of 7.1%. Although 5 patients (35.7%) died within one year after discharge from the 2nd operation, the survival rate at one year was 53.1%, which remained constant at 5 years. Importantly, none of our patients, including deceased patients, suffered from AAEs observed on CT, recurrent PVE, or PVF after hospital discharge.

Our policy is to reserve GIT for cases in which traditional reoperation is deemed unfeasible because of the extreme fragility of the aortic root and annulus caused by infection or inflammation. In GIT, it is important to reinforce the remaining tissue after debridement by applying sutures firmly to the myocardial tissue. This approach may require the sacrifice of the conduction system, which is typically preserved in standard AVR or aortic root surgery. This is an inevitable complication, depending on the extent of the lesion and debridement. In our experience, complete atrioventricular block occurred in 21.4% of cases immediately after the operation. Another potential concern with this technique is LVOT narrowing. Although this was not a particular issue in the present cases, attention must be paid to the length and thickness of the felt used.

### Limitations

This study has several limitations. The number of patients was relatively small, and the study design was retrospective and nonrandomized. Additionally, no comparative analysis with other surgical approaches was conducted in this study.

## Conclusion

The midterm outcomes of GIT are acceptable, even in the presence of serious preoperative conditions. The advantages of GIT include 1) reinforcement of the LVOT and aortic root using a unique suture technique, 2) excellent surgical view, 3) availability of various graft sizes and types, 4) prevention of graft damage, and 5) precision in suture placement. These benefits make GIT a viable option for reconstructing damaged LVOT and aortic root.

## Acknowledgments

We would like to thank Dr. Vipin Balachandran, Dr. Ayeshmanthe Rathnayake, ENAGO (www.enago.com), and Cambridge English Correction Service (www.cambridge-correction.com) for English editing.

## Declarations

### Funding

This research did not receive any specific grant from funding agencies in the public, commercial, or not-for-profit sectors.

### Authors’ contributions

All authors have read and approved the final version of the manuscript.

TN: Project administration, conceptualization, data curation, formal analysis, investigation, methodology, writing—original draft, writing—review and editing. AI: Conceptualization, project administration, data curation, methodology, validation. NI: Visualization. TU: Conceptualization, methodology, supervision, writing—review and editing. YN: Conceptualization, methodology, supervision, writing—review and editing.

### Data availability

Underlying data will be made available from the corresponding author upon reasonable request.

### Ethics approval and consent to participate

This retrospective 2-center study was approved by the Institutional Review Boards of Chiba-Nishi General Hospital and Yamagata University Hospital, Japan (approval no. TGE01987-025). Written informed consent was obtained from all patients.

### Consent for publication

Written informed consent was obtained from all patients.

### Disclosure statement

The authors declare that they have no competing interests.

## Supplementary Movie

Graft insertion technique for redo aortic root surgery.
